# Die Wechselwirkung mit ribosomalen Proteinen begleitet die Stressinduktion des Wirkstoffkandidaten BOLD‐100/KP1339 im endoplasmatischen Retikulum

**DOI:** 10.1002/ange.202015962

**Published:** 2021-02-01

**Authors:** Benjamin Neuditschko, Anton A. Legin, Dina Baier, Arno Schintlmeister, Siegfried Reipert, Michael Wagner, Bernhard K. Keppler, Walter Berger, Samuel M. Meier‐Menches, Christopher Gerner

**Affiliations:** ^1^ Institut für Anorganische Chemie Fakultät für Chemie Universität Wien Währinger Str. 42 1090 Wien Österreich; ^2^ Institut für Analytische Chemie Fakultät für Chemie Universität Wien Währinger Str. 38 1090 Wien Österreich; ^3^ Forschungsnetzwerk “Chemistry, Microbiology and Environmental Systems Science” Universität Wien Währinger Str. 42 1090 Wien Österreich; ^4^ Institut für Krebsforschung und Comprehensive Cancer Center Universitätsklinik für Innere Medizin I Medizinische Universität Wien Borschkegasse 8a 1090 Wien Österreich; ^5^ Forschungscluster “Translational Cancer Therapy Research” Universität Wien Währinger Str. 42 1090 Wien Österreich; ^6^ Großgeräteeinrichtung für Umwelt- und Isotopen-Massenspektrometrie Zentrum für Mikrobiologie und Umweltsystemwissenschaft Universität Wien Althanstr. 14 1090 Wien Österreich; ^7^ Core Facility für Cell Imaging und Ultrastrukturforschung Althanstr. 14 1090 Wien Österreich; ^8^ Joint Metabolome Facility Universität Wien und Medizinische Universität Wien Währinger Str. 38 1090 Wien Österreich

**Keywords:** Bioanorganische Chemie, Metalle in der Medizin, Multi-Omik, Ribosom, Ruthenium

## Abstract

Der metallhaltige Wirkstoff BOLD‐100/KP1339 zeigte bereits vielversprechende Resultate in verschiedenen *In vitro*‐ und *In vivo*‐Tumormodellen sowie in klinischen Studien. Der detaillierte Wirkmechanismus wurde jedoch noch nicht komplett aufgeklärt. Als entscheidende Wirkstoffeffekte kristallisierten sich kürzlich die Stressinduktion im endoplasmatischen Retikulum (ER) und die damit einhergehende Modulierung von HSPA5 (GRP78) heraus. Das spontane und stabile Addukt zwischen BOLD‐100 und menschlichem Serumalbumin wurde als Immobilisierungsstrategie ausgewählt, um einen chemoproteomischen Ansatz auszuführen, der die ribosomalen Proteine RPL10, RPL24 und den Transkriptionsfaktor GTF2I als potentielle Interaktoren dieser Ru(III)‐Verbindung identifizierten. Dieses Ergebnis wurde mit proteomischen und transkriptomischen Profiling‐Experimenten kombiniert, was die Interpretation einer ribosomalen Beeinträchtigung sowie der Induktion von ER‐Stress unterstützte. Die Bildung von Polyribosomen und begleitende ER‐Schwellungen in behandelten Krebszellen wurden zudem durch TEM‐Messungen bestätigt. Somit scheint eine direkte Wechselwirkung von BOLD‐100 mit ribosomalen Proteinen die ER‐Stressinduktion und die Modulierung von GRP78 in Krebszellen zu begleiten.

## Graphical abstract

Multi‐Omik‐Analysen zeigen, dass der tumorinhibierende Wirkstoffkandidat BOLD‐100/KP1339 gezielt mit den ribosomalen Proteinen RPL10/RPL24 sowie GTF2I wechselwirken könnte. Schwellungen des endoplasmatischen Retikulums (ER) und der Nachweis der Bildung von Polyribosomen durch bildgebende Verfahren weisen darauf hin, dass eine Behandlung mit BOLD‐100 nicht nur die Proliferation hemmt, sondern auch zu einer funktionellen Störung des ER beiträgt.

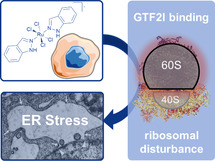

Der Rutheniumkomplex Natrium *trans*‐[Tetrachlorido‐bis(1*H*‐Indazole)Ruthenat(III)] (BOLD‐100/KP1339, Abbildung 1) ist unter den am meisten untersuchten metallhaltigen Wirkstoffen gegen Krebserkrankungen.^[1]^ Die Verbindung zeigte vielversprechende tumorhemmende Effekte in einem autochthonen Tumormodell^[2]^ und die sichere Anwendung wurde in klinischen Studien bewiesen.^[3]^ Derzeit läuft eine klinische Phase 1b‐Studie mit BOLD‐100 (*NCT04421820*). Im Gegensatz zu den klinisch breit eingesetzten platinhaltigen Wirkstoffen, die DNA‐Schäden herbeiführen, übt dieser Wirkstoff seine Effekte über andere Biomoleküle aus.^[4]^ Kürzlich stellte sich eine verringerte Expression des Glukose‐regulierten Proteins von 78 kDa (GRP78, HSPA5) in Kombination mit ER‐Stress und der Induktion von reaktiven Sauerstoffspezies (ROS) als generell akzeptierter Wirkstoffeffekt in verschiedenen 2D und 3D Tumormodellen heraus.^[5]^ Trotz weitreichender Forschungsbestrebungen blieben die Vorgänge, die ER‐Stress auslösten, sowie potentielle molekulare Ziele von BOLD‐100 nur schwer fassbar.

**Figure 1:**
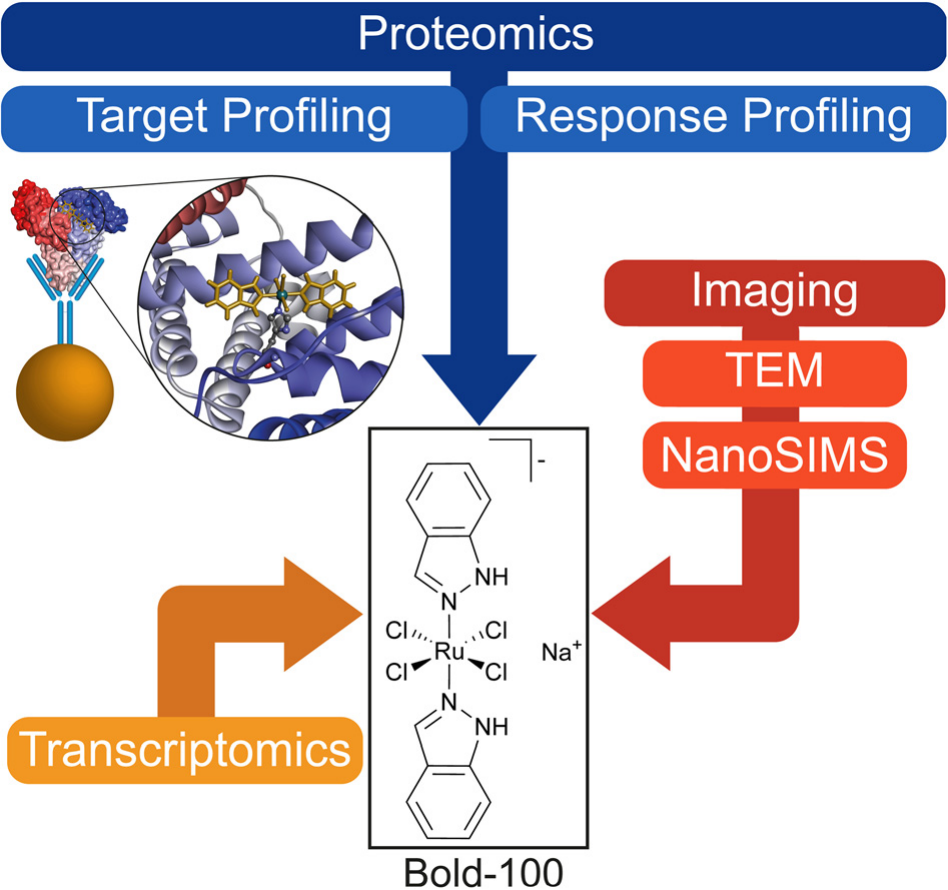
Schematischer Arbeitsablauf des Multi‐Omik Ansatzes, der in dieser Studie verfolgt wurde. BOLD‐100 wurde mit menschlichem Serumalbumin (HSA) und anti‐HSA Antikörpern auf magnetischen Kügelchen immobilisiert um chemoproteomische Verfahren (Target Profiling) zu ermöglichen. Zudem wurde der Multi‐Omik‐Ansatz durch proteomisches und transkriptomisches Profiling realisiert. Bildgebende Verfahren wurden angewendet, um die mechanistischen Vorschläge anhand der zellulären Ultrastruktur und Wirkstoffverteilung in HCT116 Krebszellen zu überprüfen.

Die Metalloproteomik benennt einen aufkommenden Ansatz um potentielle Zielmoleküle und Wirkmechanismen von metallhaltigen Wirkstoffen aufzuklären und wurde konzeptuell bereits mit anderen Omik‐Techniken in Verbindung gebracht.[^1b^, ^6^]

In diesem Bereich wurden Lösungen gefunden, um Proteinangriffsstellen von inerten metallhaltigen tumor‐inhibierenden Wirkstoffkandidaten basierend auf Gold^[7]^ oder Platin^[8]^ zu identifizieren und zu validieren. Dies wurde zudem für labile metallorganische Ruthenium‐Prodrugs erreicht.^[9]^ Ferner wurden ähnliche Strategien angewandt um Metalloproteome in lebenden Zellen zu charakterisieren.^[10]^ Die Integration von Metalloproteomik mit weiteren Omik‐Ebenen (z. B. Transkriptomisches Profiling) ist besonders vielversprechend, um neuartige Einblicke in die Wirkmechanismen von metallhaltigen Wirkstoffen zu erhalten.^[6]^

BOLD‐100 ist eine Prodrug, die durch Hydrolyse der labilen Chloridoliganden und anschließender Reduktion zu Ruthenium(II) aktiviert wird.[^1a^, ^4^] Die Oxidationsstufe +III ist dabei sogar in Geweben über längere Zeitintervalle stabil.^[11]^ Der Wirkstoffkandidat weist eine schnelle Anbindung an menschliches Serumalbumin (HSA) über nicht‐kovalente Wechselwirkungen auf, die langsam in stabile Ru‐HSA Addukte übergehen. Letztere sind durch koordinative Bindungen charakterisiert.^[12]^ Durch Röntgenbeugungsanalysen konnte gezeigt werden, dass die trans‐Indazolliganden in einigen Fälle erhalten bleiben,^[13]^ während sie anderen Berichten zufolge ausgetauscht wurden.^[14]^ Die natürliche Reaktivität von BOLD‐100 mit HSA und die Bildung von stabilen Addukten wurde bereits als Tumor‐Targeting‐Mechanismus vorgeschlagen.^[1a]^ In dieser Zuschrift wurde die Bildung dieser stabilen Addukte ausgenutzt, um BOLD‐100 mittels HSA zu immobilisieren und somit chemoproteomische Ansätze zu ermöglichen, da präparative Lösungen bisher nicht erfolgreich waren. Wir zeigen, dass BOLD‐100 direkt mit ribosomalen Proteinen wechselwirken kann und weisen dies durch Multi‐Omik Analysen in Verbindung mit bildgebenden Verfahren, wie Ultrastruktur‐auflösender Elektronenmikroskopie und hoch ortsaufgelöster Sekundärionen‐Massenspektrometrie (NanoSIMS), nach.^[15]^

Potentielle Proteinangriffsstellen von BOLD‐100 wurden durch Adaption einer bereits von uns beschriebenen Vorgangsweise mittels Shotgun Proteomik basierend auf Markierungsfreier Quantifizierung (LFQ) durchgeführt.^[16]^ Dieses Target‐Response Profiling kombiniert die Untersuchung von potentiellen Proteininteraktoren eines immobilisierten Wirkstoffes mit einer umfassenden Perturbationsstudie auf der Proteinebene. HSA wurde dazu auf magnetischen Kügelchen mit anti‐HSA Antikörpern immobilisiert und dann mit BOLD‐100 inkubiert, um gebundene BOLD‐100‐HSA Addukte herzustellen (s. Hintergrundinformation, Abbildung 1). Diese Immobilisierungsstrategie bildet auch die *in vivo* Situation des Wirkstoffkandidaten ab, der rasch an Serumproteine bindet, wie unter anderem in Tierexperimenten gezeigt werden konnte.^[17]^ Das Target Profiling wurde durch einen chemoproteomischen Ansatz unter Verwendung von nativen Gesamtzelllysaten aus HCT116 Kolonkarzinomzellen durchgeführt. Die proteinhaltigen Zelllysate wurden für 2 h mit dem immobilisierten Wirkstoff inkubiert. Nach gründlichem Waschen wurden die gebundenen Proteine unter milden Bedingungen mit Zitratpuffer eluiert und für die Analyse mittels Shotgun Proteomik prozessiert. Die Pull‐Downs wurden differentiell durchgeführt, um unspezifische Bindungsvorgänge ausschließen zu können. Nebst eines normalen Pull‐Down Experimentes wurden kompetitive Pull‐Down Experimente ausgeführt, die eine Vorbehandlung des Zelllysates mit freiem BOLD‐100 vor der Exposition mit dem immobilisierten Wirkstoff beinhaltet. Letzteres bindet selektive Bindungsstellen ab, sodass daraus ausschließlich unspezifische Bindungspartner resultieren. Durch deren Subtraktion vom Target Profil des normalen Pull‐Downs werden die unspezifischen Bindungspartner aufgehoben und es bleiben die selektiven Bindungspartner übrig.

Die Anreicherungsdarstellung zeigt das differentielle Protein Target Profil von BOLD‐100 auf (Abbildung 2 A). Die Bindungsselektivität eines Proteins wird durch den Anreicherungsfaktor zwischen normalem und kompetitiven Pull‐Down auf der *y*‐Achse wiedergegeben. Die *x*‐Achse zeigt die assoziierte Signifikanz der Anreicherung mittels p‐Werten. Das Target Profiling Experiment mit dem BOLD‐100‐HSA Addukt lieferte 57 Proteine als wahrscheinliche Interaktoren. Diese werden in der Abbildung als Kreise dargestellt, deren Größe die LFQ Intensität visualisiert (Abbildung 2 A).

**Figure 2:**
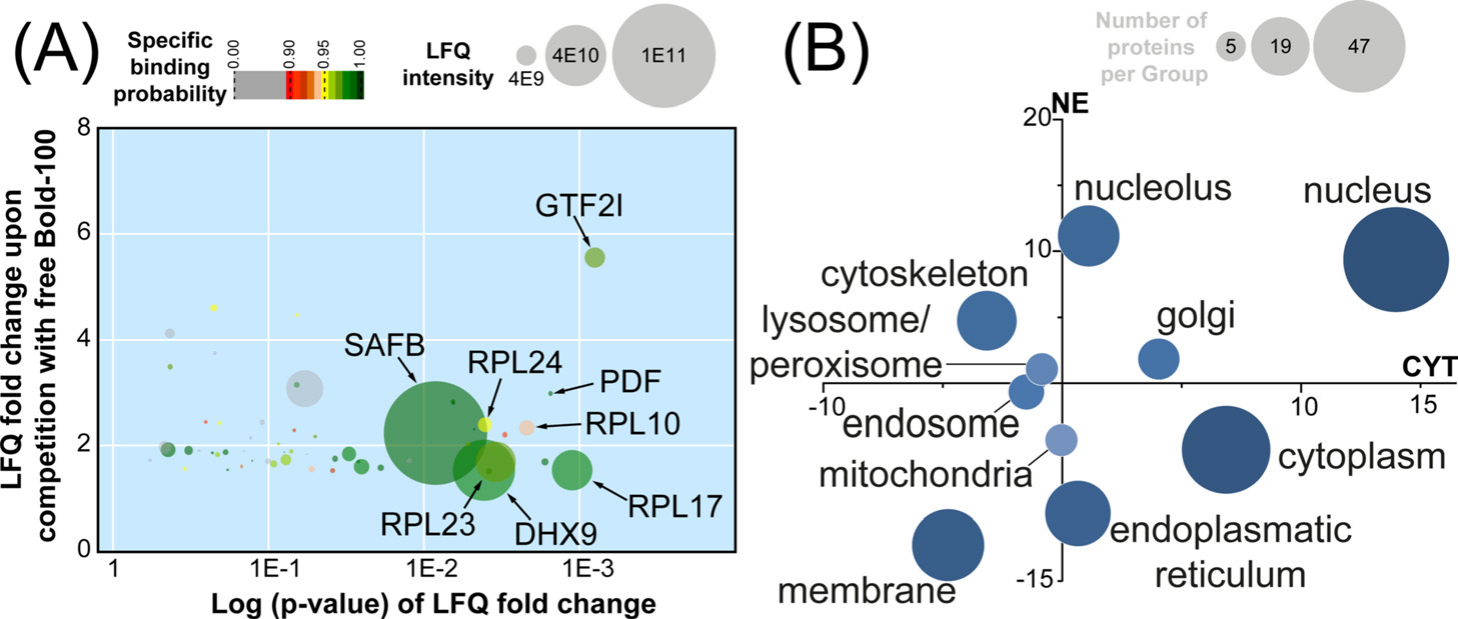
(**A**) Die Anreicherungsdarstellung zeigt potentielle Proteininteraktoren von BOLD‐100. Der Wirkstoff wurde als HSA‐Addukt immobilisiert und dann mit nativen Zelllysaten aus HCT116 Krebszellen inkubiert. Die *y*‐Achse beschreibt die LFQ Anreicherung des jeweiligen Proteins im Pull‐Down, während die assoziierten p‐Werte auf der *x*‐Achse angezeigt werden. Die Farbe weist auf die spezifische Bindungswahrscheinlichkeit hin und die Kreisgrößen visualisieren die LFQ‐Intensitäten der Proteine. (**B**) Signifikant regulierte Proteine nach Behandlung von HCT116 Krebszellen mit BOLD‐100 (80 μM, 24 h) wurden anhand zellulärer Kompartimente gruppiert und nach der summierten Expressionsänderung in zytoplasmischen (CYT, *x*‐Achse) und Kernfraktionen (NE, *y*‐Achse) abgebildet. Die Kreisgröße visualisiert die Anzahl der Proteine in der jeweiligen Gruppe.

Die Anzahl der detektierten Interaktoren ist kleiner als bei früher untersuchten metallhaltigen Wirkstoffen, was auf die Immobilisierung des Wirkstoffes mittels HSA zurückzuführen sein könnte. Proteine der großen ribosomalen Untereinheit (RPL10, RPL17, RPL23 und RPL24) bildeten eine bedeutende Gruppe im Target Profil, was eine direkte Wechselwirkung von BOLD‐100 mit ribosomalen Bestandteilen suggeriert (Abbildung S1). Als Interaktor mit dem höchsten Anreicherungsfaktor wurde der General Transcription Factor II‐I (GTF2I) identifiziert, der unter anderem die Zellantwort auf ER‐Stress reguliert, *z. B*. durch Anbindung an die Promoterregion von GRP78.^[18]^ Eine Unterbindung der Promoterfunktion von GTF2I durch Wechselwirkung mit BOLD‐100 könnte somit in direktem Zusammenhang mit der verringerten Expression von GRP78 stehen. Das Target Profil von BOLD‐100 zeigt also experimentell bestimmte Interaktoren, die deutlich im Zusammenhang mit ER Homöostase stehen.

Danach wurde ein proteomisches Response Profiling Experiment^[19]^ durchgeführt, um die Daten des Target Profilings zu ergänzen. Dafür wurden HCT116 Krebszellen mit BOLD‐100 (80 μM) für 24 h behandelt. Die zytoplasmischen (CYT) und Kernfraktionen (NE) wurden isoliert und proteolytisch verdaut, um eine Analyse mittels LFQ Shotgun Proteomik zu ermöglichen. Bei dieser Dosis wurden relativ geringe Proteomveränderungen festgestellt. Von 4193 identifizierten Proteinen (FDR=0.01 auf Peptid‐ und Proteinebene) wurden nur 116 in der zytoplasmischen und 86 in der Kernfraktion signifikant reguliert vorgefunden (FDR=0.05 und S0=0.1). Es scheint, dass BOLD‐100 das Proteom nicht im gleichen Ausmaß wie andere rutheniumhaltige Wirkstoffe beeinflusst.[^16^, ^20^] Eine Übersicht über die gesamten Proteomveränderungen durch Behandlung mit BOLD‐100 wurde durch Gruppierung von signifikant regulierten Proteinen anhand ihrer subzellulären Kompartimentierung erhalten. Die summierten Expressionsänderungen der Proteine in den jeweiligen Gruppen wurden in den Dimensionen der CYT und NE Fraktionen aufgetragen und visualisieren die zelluläre Antwort auf diese Perturbation (Abbildung 2 B). Die Kreisgröße korreliert dabei mit der Anzahl der Proteine in jeder Gruppe. Eine ausgeprägte Reduktion der Expression wurde für ER‐Proteine in der NE Fraktion beobachtet, während Kern‐ und Nukleolus‐ Proteine eine erhöhte Expression zeigten. Im Gegensatz zu anderen Ruthenium Wirkstoffkandidaten wurden mit BOLD‐100 nur geringe Expressionsänderungen von mitochondriellen Proteinen verzeichnet.^[20]^

Die potentiellen Interaktoren des Target Profilings und die Proteine mit signifikanten Expressionsänderungen des Response Profilings wurden anschließend durch eine Netzwerkanalyse mit STRING^[21]^ in Cytoscape^[22]^ ausgewertet (Abbildung 3 A und S2). Das erhaltene Netzwerk zeigt, ob potentielle Proteinangriffsstellen mechanistisch mit den Wirkstoffeffekten in Krebszellen zusammenhängen. Es wurden dabei drei biologische Prozesse gefunden, die von Bedeutung sind, nämlich die Antwort auf ungefaltete Proteine im ER (UPR, blau), ribosomale Biogenese (orange) und mRNA Prozessierung (rot). Potentielle Interaktoren sind grün markiert. Interessanterweise zeigte das Netzwerk zentral gelegene ribosomale Proteine, die ribosomale Biogenese und mRNA Prozessierung verbinden. Die Analyse untermauerte zudem die Verbindung zwischen GTF2I und HSPA5, die einen ER‐Stress Cluster verbinden. Das Target‐Response Netzwerk unterstützt demnach eine direkte Wechselwirkung von BOLD‐100 mit ribosomalen Proteinen und potentiell auch GTF2I, die die beobachteten Änderungen in ribosomaler Biogenese und ER Homöostase in behandelten HCT116 Zellen herbeiführen könnten.

**Figure 3:**
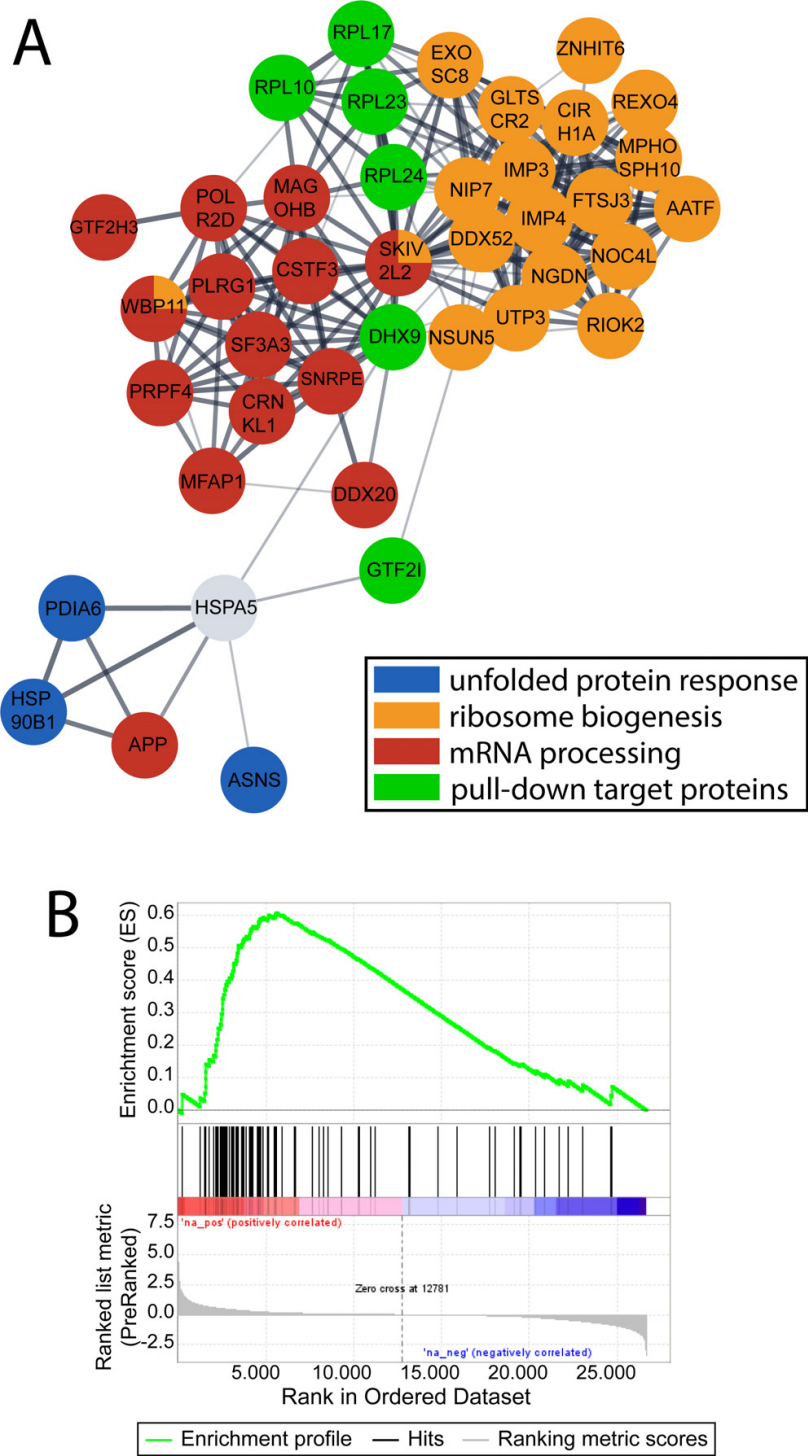
(**A**) Auszug aus der STRING Analyse der beeinflussten Proteine (FDR=0.05, S0=0.1) durch BOLD‐100 Behandlung (Cytoscape). Farben weisen auf die Zugehörigkeit zu folgenden Gene Ontology (GO) Termen hin. Blau: Endoplasmic reticulum unfolded protein response (GO.0030968); Orange: Ribosome biogenesis (GO.0042254); Rot: mRNA processing (GO.0006397); Grün: Proteine durch den Pull‐Down identifiziert wurden und HSPA5 als bekannte Zellantwort auf den Wirkstoff. (**B**) Die Gen‐Set Anreicherungsanalyse identifizierte eine signifikante Anreicherung des Terms „Ribosome“ in der KEGG Datenbank.

Die Beteiligung von ribosomalen Proteinen am Wirkmechanismus von BOLD‐100 wurde weiters auf der mRNA Ebene mittels transkriptomischem Profiling untersucht. Dafür wurden HCT116 Zellen mit BOLD‐100 (100 μM) für 6 h behandelt und die gesamte mRNA isoliert und analysiert. Der Term “Ribosome”, welcher mRNA für ribosomale Proteine enthält, wurde generell durch die Behandlung hochreguliert und zeigte einen Anreicherungswert von 0.6. Dies stellt eines der Top 16 angereicherten Gen‐Sets dar (Abbildung 3 B). mRNA von PRL10, RPL17, RPL23 und RPL24 wurde wie im Pull‐Down Experiment detektiert, jedoch wurde nur die mRNA von RPL10 und RPL24 hochreguliert vorgefunden.

Zusätzliche Evidenz für eine direkte Wechselwirkung von BOLD‐100 mit ribosomalen Bestandteilen wurde in früheren Berichten gefunden, in denen die Verteilung des Wirkstoffes in behandelten HCT116 Zelllysaten mittels Größenausschluss‐Chromatografie gekoppelt an induktiv‐gekoppelte Plasma MS untersucht wurden. In diesen Studien wurde Ruthenium vorwiegend an die hochmolekulare Fraktion (>700 kDa) gebunden vorgefunden, was eine Wechselwirkung mit großen Proteinkomplexen, wie Ribosomen, nahelegt.^[23]^

Anschließend wurden bildgebende Verfahren angewandt, um die subzelluläre Verteilung von BOLD‐100 in behandelten HCT116 Zellen aufzuklären (Abbildung 4). Dafür wurden Zellen erst als Einzelschicht kultiviert und die geernteten Zellpellets in einem niederviskosen Harz eingebettet. Es wurden Schnitte von 100 nm Dicke präpariert und analysiert. Ruthenium wurde homogen verteilt vorgefunden. Es gab keine signifikanten Unterschiede zwischen Zytoplasma, Kern oder Nukleolus (Abbildung 4 A). Das Zytoplasma zeigte einige Organellen‐ähnliche Aggregate, die eine erhöhte Rutheniumanreicherung anzeigten. Die weitgehend homogene Rutheniumverteilung in HCT116 Zellen unterstützt somit die direkte Wechselwirkung von BOLD‐100 mit zytoplasmischen Ribosomen.

**Figure 4:**
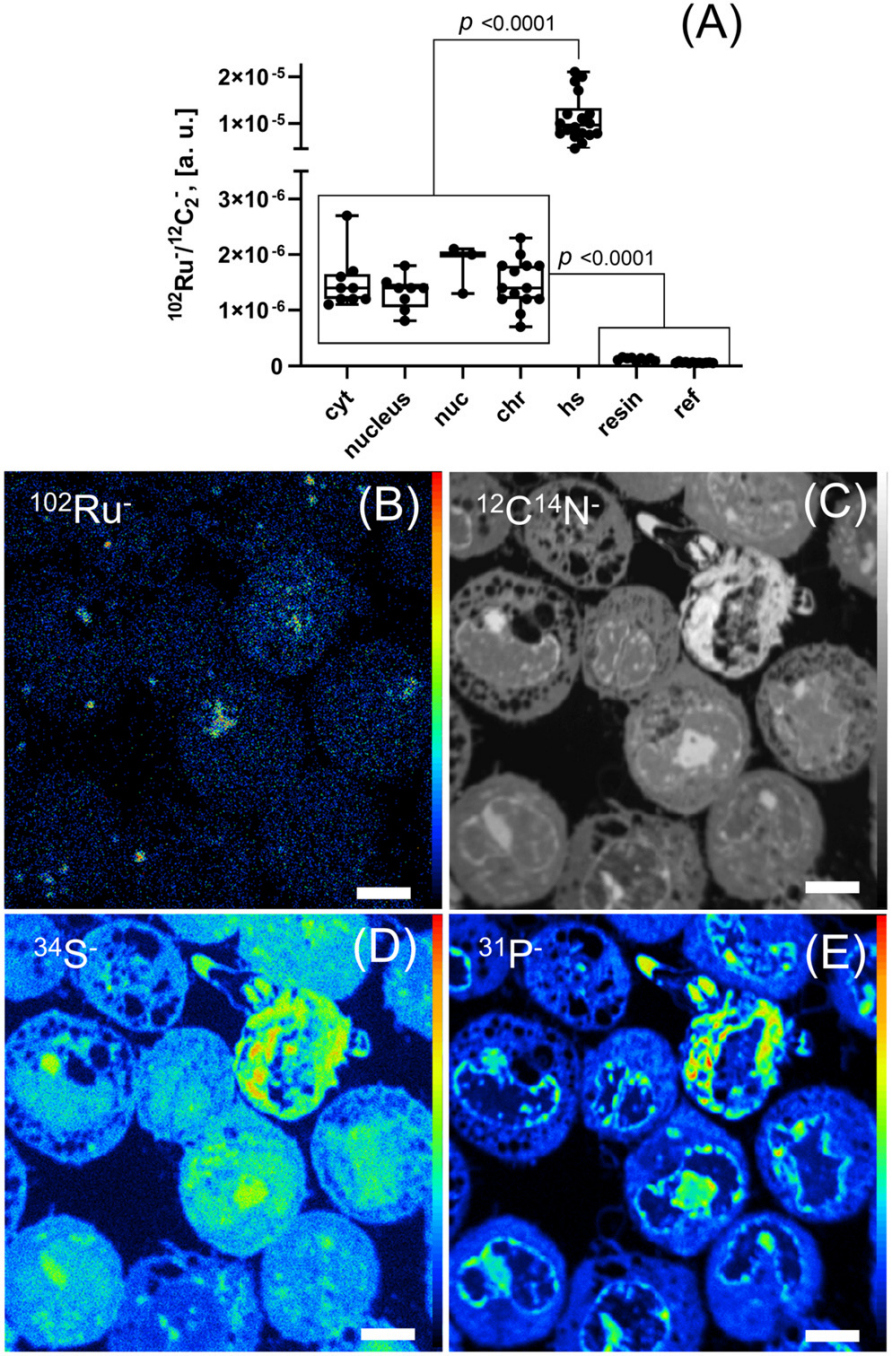
Visualisierung der Rutheniumverteilung in BOLD‐100 behandelten HCT116 Krebszellen mittels NanoSIMS. (**A**) Bestimmung des relativen Ruthenium Gehaltes in subzellulären Kompartimenten von HCT116 Zellen nach Behandlung (400 μM, 24 h) durch orts‐spezifische Auswertung von ^12^C_2_^−^ normalisierten ^102^Ru^−^ Signalintensitäten in “regions of interest” (ROI). ^102^Ru^−^ (**B**), ^12^C^14^N^−^ (**C**), ^34^S^−^ (**D**) und ^31^P^−^ (**E**) Sekundärionen‐Intensitätsverteilungsbilder, die die Anreicherung des Wirkstoffs anzeigen. Abkürzungen: cyt – Zytoplasma, nuc – Nukleolus, chr – Heterochromatinstrukturen, hs – zytoplasmische Aggregate der Ru Anreicherung, resin – Extrazelluläre Regionen (Epoxidharz), ref. – Werte einer unbehandelten Kontrollprobe. Maßstableiste: 5 μm.

Der nachfolgende 100 nm Schnitt von BOLD‐100 behandelten HCT116 Zellen im Harz wurde mittels Transmissionelektronenmikroskopie (TEM) nach Färbungsschritten mit Gadolinium Acetat und Blei Zitrat untersucht (Abbildung 5). Die TEM Bilder zeigten eine klare ER Schwellung in BOLD‐100 behandelten Zellen, die zu Ausbuchtungen des perinukleären Raumes (PS) führten (Abbildung 5 A). Diese Effekte wurden in unbehandelten Kontrollen nicht gefunden (Abbildung S3). Zudem wurden Ribosomen vermehrt vom ER losgelöst und in Clustern von Polyribosomen gefunden, die Schnur‐ähnliche Formationen ausbildeten (Abbildung 5 D, Sternchen). Die vernetzende Wirkung von BOLD‐100 auf Proteine wurde bereits früher festgestellt und unterstreicht dieses Resultat.^[24]^ Rundliche Einzelmembranhaltige zytoplasmische Organellen mit lamellaren und vesikulären Inhalten wurden weiters beobachtet. Diese Vesikel überlappen mit den Ruthenium Aggregate, die durch NanoSIMS beobachtet wurden und könnten einen Versuch der Krebszellen darstellen, die Rutheniumladung zu entgiften (Abbildung S4).

**Figure 5:**
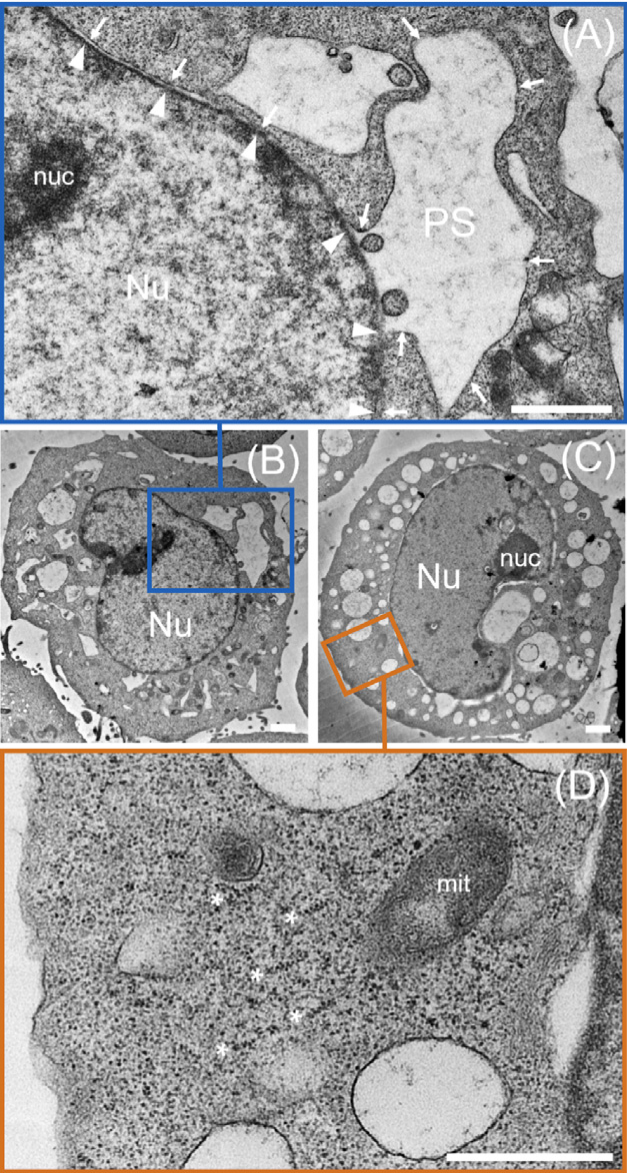
Morphologie von ER‐Stress nach Behandlung von HCT116 Krebszellen durch BOLD‐100 (400 μM, 24 h). Transmissionselektronen‐mikroskopische Darstellung von ER Schwellung, die zu Ausbuchtungen des perinukleären Raumes (PS), sowie ER Vakuolisierung führte (**A**–**D**). Die äußere Kernmembran ist durchgehend verbunden mit dem ER und durch Pfeile in **A** (Vergrößerung von **B**) gekennzeichnet, während die Pfeilspitzen die innere Kernmembran kennzeichnen. Ribosomen bildeten Aggregate (mit Sternchen markiert) und sind sichtbar im Zytoplasma **D** (Vergrößerung von **C**). Abkürzungen: Nu – Kern, nuc – Nukleolus, mit – Mitochondrien, PS – Perinukleärer Raum. Maßstableiste: 1 μm.

Zusammenfassend zeigt diese Studie durch einen Multi‐Omik‐Ansatz und bildgebende Verfahren wie NanoSIMS und TEM, die molekularen Ereignisse auf, die durch BOLD‐100 Behandlung zu ER‐Stress führen könnten. ER‐Stress wurde in früheren Studien als besonderer Wirkmechanismus dieses Ruthenium(III) Wirkstoffkandidaten identifiziert.[^1a^, ^1c^] BOLD‐100 scheint direkt mit ribosomalen Proteinen zu interagieren, wahrscheinlich mit RPL10 und/oder RPL24. Dies führt nicht nur zu einer Hemmung der Proliferation, sondern auch zu einer funktionellen Störung des ER, was sich durch die Bildung von Polyribosomen, begleitende ER Schwellungen und Vesikelbildung in Krebszellen nach Behandlung mit BOLD‐100 bei höheren Dosen manifestiert.

## Conflict of interest

Die Autoren erklären, dass keine Interessenkonflikte vorliegen.

## Supporting information

As a service to our authors and readers, this journal provides supporting information supplied by the authors. Such materials are peer reviewed and may be re‐organized for online delivery, but are not copy‐edited or typeset. Technical support issues arising from supporting information (other than missing files) should be addressed to the authors.

Supplementary
